# Dementia Rehabilitation Training for General Practitioners and Practice Nurses: Does It Make a Difference?

**DOI:** 10.3390/nursrep14040226

**Published:** 2024-10-21

**Authors:** Den-Ching A. Lee, Grant Russell, Terry P. Haines, Keith D. Hill, Claire M. C. O’Connor, Natasha Layton, Kate Swaffer, Marita Long, Catherine Devanny, Michele L. Callisaya

**Affiliations:** 1 Rehabilitation Ageing and Independent Living (RAIL) Research Centre, School of Primary and Allied Health Care, Faculty of Medicine, Nursing and Health Sciences, Monash University, Frankston, VIC 3199, Australia; keith.hill@monash.edu (K.D.H.); natasha.layton@monash.edu (N.L.); 2 National Centre for Healthy Ageing, Monash University and Peninsula Health, Frankston, VIC 3199, Australia; terry.haines@monash.edu (T.P.H.); catherine.devanny@monash.edu (C.D.); michele.callisaya@monash.edu (M.L.C.); 3 Department of General Practice, School of Public Health and Preventive Medicine, Faculty of Medicine, Nursing and Health Sciences, Monash University, Melbourne, VIC 3004, Australia; grant.russell@monash.edu; 4 School of Primary and Allied Health Care, Faculty of Medicine, Nursing and Health Sciences, Monash University, Frankston, VIC 3199, Australia; 5 School of Psychology, University of New South Wales, Sydney, NSW 2052, Australia; claire.oconnor@unsw.edu.au; 6 Hammond Care, Centre for Positive Ageing, Sydney, NSW 2170, Australia; 7 Neuroscience Research Australia, Sydney, NSW 2031, Australia; 8 Department of Sociology, School of Justice and Society, University of South Australia, Lorne Avenue, Magill, SA 5072, Australia; kate.swaffer@mymail.unisa.edu.au; 9 Department of General Practice and Primary Care, Melbourne Medical School, University of Melbourne, Parkville, VIC 3052, Australia; maritalong@gmail.com; 10 Menzies Institute for Medical Research, University of Tasmania, Hobart, TAS 7000, Australia

**Keywords:** general practitioner, practice nurse, dementia, rehabilitation, referrals, knowledge, confidence, attitudes, training, barriers

## Abstract

Background/Objectives: Rehabilitation helps reduce disability in dementia. The Australian National Dementia Action Plan identifies a gap in clear treatment pathways post-diagnosis, affecting the quality of life for those with dementia. This study assessed the impact of a one-day dementia training course and follow-up on GPs’ and practice nurses’ knowledge, attitudes, and confidence regarding dementia rehabilitation. Methods: The training, led by two experienced GPs and an academic physiotherapist, covered dementia diagnosis, allied health roles, care planning, and referrals. The follow-up involved applying the learnt material and completing a reflective task. Three longitudinal surveys (Dementia Knowledge Assessment Scale—DKAS, General Practitioners’ Attitudes and Confidence towards Dementia Survey—GPACS-D, and Dementia Rehabilitation Scale) and Likert-scale statements were conducted pre-course, post-course, and at four-month follow-up, alongside a focus group. Descriptive and regression analyses were applied to survey data, and content analysis was used for focus group data. Results: Seventeen participants (14 GPs, 3 nurses) completed the pre–post-course survey, with eight (6 GPs, 2 nurses) participating in follow-up and focus group discussions. Post-course, DKAS scores increased by 12.1%, GPACS-D by 10.1%, and the dementia rehabilitation scale by 9.4%. Likert-scale statements improved by 8–79%. At the four-month follow-up, there was a slight, non-significant decline in most measures. Focus groups highlighted the training’s impacts, useful components, barriers, and suggestions for improvement. Conclusion: Training GPs and practice nurses in dementia rehabilitation enhances knowledge, awareness, and confidence. Ongoing efforts are needed to sustain benefits and address referral barriers for better access to dementia rehabilitation services.

## 1. Introduction

Dementia constitutes a significant source of disability. The World Health Organisation (WHO) extends the definition of dementia beyond its biomedical definition, describing it as “one of the major causes of disability and dependency among older people globally” [1]. Rehabilitation, according to the WHO, is “a set of interventions aimed at optimising functioning and reducing disability in individuals with health conditions in interaction with their environment” [2], encompassing chronic conditions such as dementia [3]. The WHO’s global action plan emphasises the need for efficient and coordinated care pathways, incorporating rehabilitation to comprehensively address disability associated with dementia [4].

Despite strong evidence supporting the efficacy of rehabilitation treatments (or sometimes referred to as reablement) for dementia [5,6,7,8,9], individuals diagnosed with dementia are often not routinely offered rehabilitation to address associated symptoms causing disability, activity restrictions, or impairments in participation [10,11,12]. This is in contrast to accumulating evidence for therapies such as physiotherapy [13], occupational therapy [14], speech therapy [15], exercise [6], and cognitive rehabilitation [16], which have shown positive outcomes, especially when administered early in the condition. These therapies fall under the broader category of ‘allied health’, which refers to a group of healthcare professionals distinct from medical doctors and nurses. Allied health professionals provide therapeutic and diagnostic services aimed at improving the function, independence, and quality of life for individuals with a variety of health conditions. Key allied health disciplines involved in dementia care include physiotherapists, who work on improving physical function and mobility; occupational therapists, who assist individuals with maintaining independence in daily activities; speech pathologists, who address communication and swallowing difficulties; and exercise physiologists, who design programs to enhance strength and endurance. In Australia, guidelines [17] and position statements [18] advocate for referral to allied health to help individuals with dementia live well and maintain independence. However, the Australian National Dementia Action Plan Consultation Paper [19] notes a lack of clear pathways or treatment plans post-diagnosis, hindering efforts to help people live as well as possible.

General practitioners (GPs) are key primary healthcare providers for people with dementia, playing a crucial role in referring patients to allied health professionals. Practice nurses support GPs by assisting with patient assessments, coordinating referrals, and contributing to quality improvements. The Royal Australian College of General Practitioners (RACGP) Silver Book [20] emphasises non-pharmacological management to support dignity and independence. Recognising the vital role of GPs and practice nurses in dementia care, Dementia Training Australia (DTA), a consortium funded by the Australian Government provides nationwide education and training on the care of people living with dementia. DTA has run dementia education for GPs and practice nurses through regional Primary Health Networks (PHNs), with a focus on improving the knowledge and skills of the GP workforce in supporting individuals with dementia and their care partners. In addition, in the 2021–2022 budget, the Commonwealth Government provided funding to the PHNs to create local dementia pathways.

This study is part of a larger project investigating solutions to improve access to rehabilitation for people living with dementia. Phase one of the project involved co-design workshops with health professionals, people living with dementia and care partners, and representatives from organisations who support people with dementia. These workshops identified a gap in GP knowledge about the role of allied health and rehabilitation, which may result in people with dementia not being referred for these services [21]. Therefore, the aims of this study were (1) to evaluate the impact of a dementia training course, enhanced with additional content on allied health and dementia rehabilitation, on GP and practice nurses’ knowledge, attitudes, and confidence regarding dementia and dementia rehabilitation and (2) to obtain information on the usefulness of the course and barriers and facilitators to putting what was learnt into practice.

## 2. Materials and Methods

### 2.1. Design

**Figure 1:**
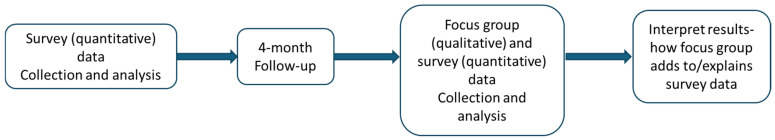
Sequential mixed-method design.

This sequential mixed-method study began with two quantitative surveys (pre and post the training day), followed by qualitative focus group discussions and a repeat survey at four months. This approach was used to assess changes in knowledge, attitudes, and confidence over time with a longitudinal design and to facilitate a deeper understanding of the topic [22] (Figure 1). Throughout the study, the checklist for mixed-method research manuscript preparation was followed [23], ensuring comprehensive and accurate reporting of the research findings.

### 2.2. Participants

Participants were GPs and practice nurses situated within a PHN in Melbourne, Australia. Inclusion criteria were the ability to attend a one-day in-person dementia training course and agreement to complete the evaluation survey both before and after the training. Participants were encouraged to opt-in for an additional follow-up learning activity and participation in a focus group and a follow-up survey. The follow-up learning activity encompassed putting into practice what was learnt at the training and a clinical reflection task. Participants were recruited via PHN channels including their website, e-newsletters, and social media. They were offered a gift voucher of AUD $150 for completing the surveys and $150 for the focus group.

Human research ethics approval was obtained prior to recruitment (Monash University project ID: 38423), and all participants provided written consent.

### 2.3. Intervention

#### 2.3.1. Dementia Training Course

The six-hour dementia training course was led by two experienced GP educators specialising in dementia, along with an academic physiotherapist (lead researcher of the project). The course aimed to enhance GPs’ dementia diagnostic and management skills and provided additional information on the roles of allied health professionals in dementia and dementia rehabilitation. The training employed interactive methods, including videos of GPs making the diagnosis and referrals, case studies, and discussion. Approximately half of the day covered the role of allied health and evidence-based strategies for optimising functioning using five structured domains: (a) cognition, (b) function, (c) psychiatric, (d) behaviour, and (e) physical [24], as well as writing personalised care plans based on the domains and making appropriate referrals.

#### 2.3.2. Follow-Up Learning Activity

The optional self-paced learning activity included the following components: (1) improving familiarity with local Dementia HealthPathways™—a website hosted by the PHNs offering clinical and referral information for conditions like dementia, including allied health professionals [25,26]; (2) creating care plans and referrals for up to three dementia patients based on the training; (3) reflecting on changes in practice following the training; and (4) identifying barriers and enablers, along with potential solutions to address the barriers. For complete details of the follow-up learning activity, see Supplementary Material, Table S1.

### 2.4. Outcome Measures

#### 2.4.1. Surveys

The surveys were administered pre- and post-course and at four months (for those who participated in the optional follow-up learning activity). Paper-based surveys were used on the training day, and online surveys (Qualtrics survey software https://www.qualtrics.com) were used at the four-month follow-up.

The survey consisted of predominantly closed-ended questions across six sections: (a) demographics and clinical practice, (b) knowledge of dementia, (c) attitudes and confidence towards dementia, (d) knowledge of dementia rehabilitation, (e) four Likert-scaled statements of knowledge and confidence related to allied health referrals, and (f) a visual analogue scale measuring confidence in understanding allied health professional roles for dementia. More details on each are as follows:

1. Questions on demographics and clinical practice included age, gender, professional roles, primary workplace, years of practice, and number of people with dementia they had treated in the last 12 months.
2. Dementia knowledge was evaluated using the Dementia Knowledge Assessment Scale (DKAS) [27], a reliable and validated 25-item scale designed to measure knowledge about dementia. The scale comprises four subscales, delineating knowledge characteristics across four domains: (a) causes and characteristics, (b) communication and behaviour, (c) care considerations, and (d) risks and health promotion. The DKAS yields a total summative score ranging from 0 to 50. Each item is scored, with 0 denoting an incorrect response to a factually true or false statement and 2 indicating a correct response. Subscale scores and the total score provide insights into the depth of understanding regarding specific aspects of dementia knowledge and overall dementia knowledge, respectively. A higher score signifies a more comprehensive understanding of the subject.
3. Attitudes and confidence towards dementia were assessed using the General Practitioners’ Attitudes and Confidence towards Dementia Survey (GPACS-D) [28]. This 15-item scale is a reliable and validated tool that measures GP’s confidence and attitudes in relation to dementia. The survey encompasses three subscales, namely (a) Attitude to Care, (b) Engagement, and (c) Confidence in Clinical Ability. Participants rated each item on a Likert scale, where 1 indicated “strongly disagree”, 3 “neither agree nor disagree”, and 5 “strongly agree”. Subscale average scores range from 1 to 5, and the total average score ranges from 3 to 15. Higher subscale and total scores signify more positive attitudes and greater confidence regarding the specific aspect of dementia care and overall dementia care, respectively.
4. Perceptions regarding rehabilitation for individuals with dementia were assessed using a specially designed 16-item dementia rehabilitation scale questionnaire. It was developed by the research team and informed by current evidence [5,6,7,8,9] (Appendix A). Participants rated each item on a Likert-scale ranging from 1 to 5 (1 indicating “strongly disagree”, 3 “neither agree nor disagree”, and 5 “strongly agree”). The total summative score, indicative of perception strength, spans from 16 to 80. A higher score reflects a more positive perception towards dementia rehabilitation.
5. Agreement with four Likert-scaled statements: (1) Much can be done to support people with dementia to maintain their independence in everyday activities; (2) I know which allied health professionals in my area provide therapy for people with dementia to help them maintain their independence for as long as possible; (3) I feel confident to discuss dementia reablement and rehabilitation therapies with my patients with dementia; (4) I feel confident my referrals to health professionals will be accepted for people living with dementia. Participants rated each item on a Likert-scale ranging from 1 to 5 (1 indicating “strongly disagree”, 3 “neither agree nor disagree”, and 5 “strongly agree”). These statements were developed based on barriers identified during Phase 1 co-design workshops [21] and were informed by the General Practitioners’ Attitudes and Confidence towards Dementia Survey (GPACS-D) [28], with new items specifically designed to address dementia rehabilitation.
6. Confidence in understanding allied health professional roles for dementia. Ratings range from 0 to 10 (0 = I know nothing, 10 = I know very well). A higher score signifies more confidence in understanding the roles of allied health professionals.

#### 2.4.2. Focus Groups

Two online focus groups took place to accommodate preferred times and were conducted four months after the training course. MLC and ML, both of whom served as trainers for the training course, facilitated the focus groups. The sessions took place on the Zoom virtual meeting platform and lasted for 60 min. The discussion was recorded and transcribed verbatim using otter.ai and cross checked by MLC.

Semi-structured questions encompassed a range of topics, including (1) what participants had learnt from the training course and follow-up activity, (2) any changes to practices, (3) barriers and enablers to referral to allied health, and (4) any further training or solutions they believed would assist them in the future with allied health or rehabilitation referrals.

### 2.5. Data Analysis

Descriptive statistics were used to present demographic and clinical practice information. Mean (SD) scores for the questionnaires, Likert-scaled statements, and the confidence ratings were calculated pre- and post-course and at the four-month follow-up. Relative changes (in percentages) in the mean scores were also calculated.

Linear regression analysis with data clustered by individual participant utilising robust standard error [29] were employed to examine the absolute differences over time between continuous outcome variables (DKAS, GPACS-D, and dementia rehabilitation scale questionnaire), and ordinal logistic regression was used for ranked outcome variables (Likert-scale statements and confidence in understanding allied health professional roles for dementia). Statistical analyses were conducted using STATA SE version 15.1 (StataCorp. 2017, College Station, TX, USA), with an alpha criterion set at *p* < 0.05.

Qualitative data from focus group discussions were analysed using content analysis [30], a method for identifying and quantifying the presence of specific words, themes, or concepts within qualitative data. This approach allows researchers to examine the frequency, meaning, and relationships between these elements. The steps undertaken for the content analysis were as follows:

(1) Independent review: DCAL and MLC independently reviewed the focus group transcription to identify units of meaning within the qualitative data.
(2) Definition of meaning units: both researchers defined specific units of meaning, which are phrases, words, or concepts that carry significant relevance to the research objectives.
(3) Category establishment: based on these units of meaning, they established preliminary categories for coding the data.
(4) Collaboration: DCAL and MLC collaborated to refine and agree upon the categories, ensuring they accurately represented the data.
(5) Disagreement resolution: any disagreements in categorisation or coding were resolved through consultation with TPH.
(6) Final coding: once consensus was reached, the final categories were applied to the data, quantifying the presence and relationships of the identified elements.

## 3. Results

### 3.1. Pre and Post-Course Surveys

Seventeen participants (14 GPs and three practice nurses) attended the dementia training course. Participants had a mean age of 51.9 years (SD = 9.3), 13 (76.5%) were females, and the mean years of practice was 21.4 (SD = 11.5). Thirteen (76.5%) worked primarily in general practices, two in residential aged care facilities, and two in community health services (each 11.8%). All participants reported having treated at least one person with dementia in the past 12 months but only 10 (58.8%) had referred individuals with dementia to allied health professionals. Among these referrals, physiotherapy and occupational therapy were the most frequently referred disciplines, with 90% and 80% respectively. Further demographic details are provided in Table 1.

Pre- and post-mean and percentage changes in each outcome variable are provided in Table 2. The scores for the DKAS, GPACS-D, and the dementia rehabilitation scale questionnaire increased by 12.1%, 10.1%, and 9.4%, respectively. Notably, the most substantial improvement within DKAS was observed in the “Cause and Characteristics” domain, showing a 31.9% increase. For the GPACS-D subscale, the highest increase was in the “Confidence in Clinical Ability” category, with a 37.2% rise. However, a minor decline of 7.7% was noted in the “Engagement” category.

For Likert-scale statements pertaining to allied health knowledge and confidence, the most notable improvement was observed in the statement, “I feel confident to discuss dementia reablement and rehabilitation therapies with my patients with dementia”, showing an increase of 78.6%. Additionally, there was a rise of 24.9% in the mean confidence score regarding the understanding of the roles of allied health professionals in dementia care.

These improvements were further supported by significant absolute changes in all outcome measures, as indicated by regression analyses. The coefficients (95% confidence interval) were as follows: DKAS [5.1, 95% CI (3.2, 6.9)], with notable gains in the subscales “Causes and Characteristics” and “Risk and Health Promotion”; GPACS-D [1.1, 95% CI (0.4, 1.7)], particularly in the subscale “Confidence in Clinical Ability”; and the dementia rehabilitation scale [6.4, 95% CI (4.4, 8.5)]. Additionally, all Likert-scale statements related to allied health knowledge and confidence, as well as confidence in understanding allied health roles for dementia [2.4, 95% CI (0.8, 3.9)], showed significant improvements.

### 3.2. Four-Month Survey and Focus Group

Eight participants (6 GPs and 2 practice nurses) opted in for the follow-up self-paced learning activity. After engaging with the self-paced training, participants attended the focus groups and completed the four-month follow-up survey. Both the focus groups and survey were conducted after the training to capture participants’ reflections on the learning experience and its impact on their practice. Participants had a mean age of 53.3 years (SD = 8.7), and seven (87.5%) were female. Six participants (75%) worked primarily in GP clinics, while two (25%) worked in residential aged care facilities.

When comparing post-course assessments to the four-month follow-up, a slight decline in nearly all measured aspects was observed, indicated by relative percentage changes (Table 2). The mean scores for the DKAS and the dementia rehabilitation scale questionnaire decreased by 5.3% and 2.5%, respectively. However, two areas showed improvement: the mean scores for the GPACS-D increased by 3.3% at follow-up, and there was a 9.5% improvement in the mean Likert-scale ratings for the statement, “I feel confident to discuss dementia reablement and rehabilitation therapies with my patients with dementia”. Despite these changes, none were statistically significant when examining the absolute changes indicated by coefficients (95% confidence interval) in the outcome measures.

**Figure 2:**
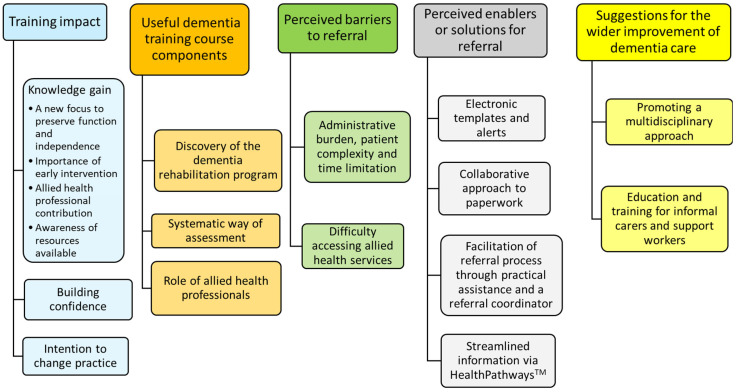
Content analysis of focus group data.

Content analysis of the qualitative data from the focus group discussions identified five main categories and a range of subcategories (Figure 2).

They are presented below, together with supportive quotes.

#### 3.2.1. Training Impact

##### Knowledge Gain

Participants gained knowledge of the importance of rehabilitation to preserve function and independence, the role of allied health professionals in dementia rehabilitation, and of resources available to support patients and families.

A new focus to preserve function and independence

“*I think my take away for that day was that it’s important to preserve the patient’s functions….it’s important to preserve their functions so that we can still emphasise on what they can still do and not what they cannot do. And trying to make some changes to the environment, to compensate for the lack of their abilities…*”

(id 8, GP)

“*just to make things functional…, you know, the special clocks and the orientation, devices, all those things. Adaptive clothing. …Keep them as independent as long as possible with what they can do, because otherwise they just decondition and quality of life goes down*.”

(id 15, GP)

Importance of early intervention

“*…the importance of early intervention to optimise people’s quality of life. Approaching diagnosis with a rehab (rehabilitation) focus positive….*”

(id 14, GP)

Allied health professional contribution

“*….. my main learning with the program was just with regards to Allied health and how we could kind of get them more involved in the care for a patient, that was really a big eye opener for me...*”

(id 8, GP)

Awareness of resources available

“*Increased awareness of resources available in community to support clients and families..*”

(id 14, GP)

“*…utilise local health networks resources.*”

(id 17, GP)

##### Building Confidence

All participants expressed increased confidence in seeing people with dementia in their practice, including referring them to appropriate services and assisting both patients and families through the process.

“*It’s given me a bit more confidence….if someone’s coming with concerns about memory loss, I know how I can approach them.*”

(id 8, GP)

“*I’ve already changed in that I’m more confident in actually referring to them (allied health)…liaising with other departments. [I] also feel more comfortable in helping the family and the patient in regards to the processes that are occurring… So I’m spending much more time because I’m confident to talk to them about that and make them feel more equipped. Now I understand, and also that I don’t need to be as frustrated, I know what to do.*”

(id 2, GP)

“*I suppose it kind of encouraged me a lot about dementia. I’ve worked in the area for years, and I think resources have fluctuated a bit, and sometimes not been as reliable. But I think now things are really much more reliable to be able to refer families to. So that was encouraging*.”

(id 14, practice nurse)

##### Intention to Change Practice

Three participants reported engaging in the follow-up learning activity by reflecting on and reviewing care pathways. However, none had developed comprehensive care plans or made referrals for their dementia patients, primarily due to the absence of suitable patients during the four months following the training. Despite this, many participants expressed a commitment to referring patients to dementia rehabilitation, altering their approach to care plans, and actively involving allied health professionals in patient care.

“*if I refer the patient to the dementia rehab (rehabilitation) programme. I think that’s an excellent program and then I’m sure that will help the patient.*”

(id 1, GP)

“*I do make much more of an effort, I really try and link people in with allied health and activities involving social connection.*”

(id 14, GP)

“*It’s got me thinking about the way I do my care plan. ….I’ve actually got a little peer group learning that I do with a couple of other people in aged care….it is contribution to care plans and how we can improve them….And I was thinking that I will structure it more around the domains for my dementia residents so that I get it right…. Yeah. I think it’s just getting everyone thinking that way ….. we should be thinking about all facets.*”

(id 15, GP)

#### 3.2.2. Useful Dementia Training Course Components

Participants highlighted several useful components of the dementia training course, including awareness of available support programs for people living with dementia, such as the recently launched 12-week home-based dementia rehabilitation program, which is exclusive to a single PHN area. Other notable components were the structured assessment of domains through case studies, and the detailed section on the role and scope of allied health in addressing each domain.

##### Discovery of the Dementia Rehabilitation Program

Participants talked about the benefit of knowing about the dementia rehabilitation program in that PHN area, and how it made it easy and quick to know where to refer to.

“*I think the most important part for me, is that I found out that there’s a dementia rehab programme. Because I looked at it, and it seems like it’s made a big difference, because if you didn’t have that, you’d have to find all the different allied health professionals interested in dementia..... I prefer that rather than I send the patient to here and then everywhere.*”

(id 1, GP)

##### Systematic Way of Assessment

Learning a systematic way to assess the patient with examples was seen as useful by participants.

“*Systematic way of assessment—the template of the 5 domains... and the driving assessment was useful.*”

(id 15, GP)

“*I found the (patient assessment) example really useful… so I would refer back to that quite a bit.*”

(id 3, GP)

##### Role of Allied Health Professionals

The section on the role of allied health professionals in dementia care was seen as very useful.

“*the potential role for OT (occupational therapy) that I hadn’t thought about…..how I utilise the services I’ve got within aged care better and what I can ask the allied health…to do...more specific in what I was asking them to do.*”

(id 15, GP)

#### 3.2.3. Perceived Barriers to Referral

Participants identified several barriers to making referrals to allied health, including time constraints during appointments, patient complexity, and the paperwork burden associated with the referral process.

##### Administrative Burden, Patient Complexity, and Time Limitation

“*It is very complicated when you have, the patient with dementia and the other needs, and then you have family members. And then you also want to maximise care, and all their chronic if not acute illnesses. And also knowing the advanced care planning and all of that on top of it. And all the legalities that go with preparing the family or the patient and their family to be able to navigate…*”

(id 2, GP)

“*We don’t have enough time in the consultation. And you know they come in with many other different reasons as well. Nurses can do paperwork for us for health assessments and care plans. But still we have to review that, and making sure that all the accurate information is in there.*”

(id 17, GP)

##### Difficulty Accessing Allied Health Services

Additionally, participants noted that the current system makes it challenging for individuals diagnosed with dementia to access services, particularly if they are not considered high risk in specific areas.

“*the way the system works is someone having a diagnosis of dementia unless they’re high risk in a particular area falls risk or whatever. It’s very, very difficult to get them seen. A lot of wait lists are closed.*”

(id 14, GP)

#### 3.2.4. Perceived Enablers or Solutions for Referral

Participants identified several potential enablers for improving the referral process to allied health. Some suggested solutions included the implementation of electronic medical record templates and alerts or adopting a collaborative approach to referral paperwork to distribute the process more evenly.

##### Electronic Templates and Alerts

“*So I’ve often wondered if we could standardise it in any way in the health records. Say we mentioned dementia. Something comes up on the screen and says, have you referred?*”

(id 3, GP)

“*I think we need to have like a standardised (referral) template….Try and make it quick also. And also if we have resources that we call….*”

(id 10, Practice nurse)

##### Collaborative Approach to Paperwork

“*this paperwork needs to be done, and let’s all do it together….Perhaps there are certain sections that we can all do just to minimise the load that’s placed on one particular person.*”

(id 3, GP)

##### Facilitation of Referral Process through Practical Assistance and a Referral Coordinator

Participants also expressed the need for practical assistance, such as having easy access to information about available allied health professionals in their area. They believed that having this information readily available would assist GPs in making appropriate referrals and coordinating care effectively.

“*Whether you’ve got the network established to make that work in practice? That’s where you go, and there’s the list of all the services. That makes a big difference in GP land. Establishing a centralised point of contact and a comprehensive list of available services…. This centralised resource would make it easier for GPs to navigate the range of services available for their patients with dementia.*”

(id 15, GP)

“*I think it’s really important to have someone kind of overseeing that process of introducing services. you know, at the client’s pace or the family’s pace.*”

(id 14, GP)

##### Streamlined Information via HealthPathways^TM^

“*I used to have access to HealthPathways^TM^ to the other side of Melbourne, and I’d use it all the time, cause it’s so. It’s streamlined, and it’s easy to access.*”

(id 15, GP)

#### 3.2.5. Suggestions for the Wider Improvement of Dementia Care Training

Participants offered several suggestions for improving training in dementia care for the wider community.

##### Promoting a Multidisciplinary Approach

Encouraging a multidisciplinary approach to dementia care was emphasised. This approach involves promoting collaboration among all healthcare professionals in managing dementia, recognising that it requires input from various specialties.

“*….I was thinking that I will structure it more around the domains for my dementia residents so that I get to be right, something specific on each of those lines. When I’m doing my upgraded care plan template. Yeah. I think it’s just getting everyone thinking that way….. what can we do for you know, that there’s multiple facets to it…And that, we should be thinking about all facets...*”

(id 15, GP)

##### Education and Training for Informal Carers and Support Workers

There was a call for education and training programs tailored to support workers and informal carers, enabling them to better assist individuals with dementia and their families.

“*be able to receive education to support their loved one….training for support workers would be really helpful around dementia.*”

(id 14, GP)

## 4. Discussion

This study evaluated the efficacy of a GP and practice nurse dementia training course and subsequent follow-up learning activities, focussing on rehabilitation therapies for managing dementia-related disabilities. Participants showed improved knowledge, confidence, and attitudes towards dementia care, supported by significant changes in outcome measures immediately after the training course. Four months later, there were slight declines in DKAS and dementia rehabilitation scale questionnaire scores but increases in GPACS-D and confidence to discuss rehabilitation therapy for people with dementia, though not significantly. Focus group discussions provided deeper insights, highlighting impacts of the training, useful components, barriers, enablers, or solutions for referrals, and suggestions for improvement. Overall, the training improved knowledge and confidence, but the follow-up showed challenges in implementing the gained knowledge into practice. This suggests a need for further strategies, support, and health system level changes to achieve real practice change.

Participants who opted in for the follow-up learning activity expressed benefits such as knowledge gain, increased confidence, and intentions to change practice. Various behavioural change models could provide insight into these findings. The positive outcomes may serve as precursors for behavioural change, akin to the contemplation and preparation stages in the Transtheoretical Model of Behavioural Change [31]. Utilising the COM-B model of behavioural change (where ‘C’ represents Capability, ‘O’ represents Opportunity, and ‘M’ represents Motivation-key components required for behaviour ‘B’ to change) [32], the dementia training aimed to enhance individual participants’ capability by augmenting knowledge and providing resources to facilitate referrals, along with hands-on practice, such as creating a GP dementia care plan, identifying local allied health service providers, and exploring funding avenues for allied health rehabilitation. It instilled motivation through cognitive processes guiding referral behaviour, such as employing reflection activities to enhance learning experiences, assess changes in practice, identify challenges, and evaluate the usefulness of the learning activity for patients. Further strategies could include academic detailing, which is increasingly utilised in general practice to enhance professional practice by providing unbiased, evidence-based information, primarily regarding medications but also emerging in the diagnosis of dementia to improve patient care [33]. This approach involves one-on-one discussions between a trained health professional (known as the academic detailer) and GPs at their workplace, employing methods originally developed by the pharmaceutical industry. Although academic detailing may also be utilised for improving practice in other areas such as dementia care, its implementation may be limited by cost. A recent systematic review showed that academic detailing alone or combined with audit and feedback alone is ineffective without intensive follow up [34].

The findings indicate that even if contemplation and motivation can lead to future actions, there are a number of barriers to be addressed, such as reducing the administrative burden associated with referrals and tackling system barriers such as improving access and availability of allied health services. The RACGP has recognised paperwork as a leading cause of GP burnout [35], as well as acknowledging the mounting pressures on GPs due to waiting list situations for specialty services [36]. A newly funded specialised dementia rehabilitation program in Melbourne, Australia, offers a free 12-week, home-based, multidisciplinary allied health therapy for people with dementia within a PHN area [37]. This program simplifies the referral process for GPs, requiring minimal paperwork. However, the availability of such programs remains limited across Australia. Importantly, these barriers to accessing dementia rehabilitation represent only a fraction of the comprehensive conceptual framework proposed by Levesque et al. for accessing healthcare, including rehabilitation [38]. This framework encompasses various determinants and barriers at multiple levels, including health systems, organisations, patients, and families, not solely GPs or allied health service providers. For example, patient factors such as having private insurance, availability of transportation to allied health services, or presence of a carer also play significant roles [39].

Participants suggested numerous avenues for resource and practice improvement, primarily aimed at reducing barriers encountered in referral practices. Streamlined resources, such as HealthPathways^TM^, having a referral coordinator, creating medical record alerts, and standardising referral template, were among the suggested solutions to alleviate administrative burdens and enhance ease of referrals. Best practice alerts in electronic health records (EHRs) have been recommended for improving quality, safety, and efficiency in primary healthcare [40]. However, a US-based study found their effects on work burden in community-based primary care practices differed for clinicians compared to support staff [40].

Effective referral systems are crucial for optimal healthcare delivery, but various factors can affect their performance [39]. While having a referral coordinator may improve efficiency and patient-centeredness, other factors can influence referral outcomes. For instance, dementia navigators and case managers in other countries have shown varying impacts. However, the diverse models and qualifications of navigators make it challenging to determine the most effective components [41,42,43]. Additionally, practice nurses play a crucial role by assisting with patient assessments, coordinate referrals, and manage follow-ups, which can impact the overall efficiency and effectiveness of the referral system [44].

This study’s strengths lie in its longitudinal follow-up design, offering data on knowledge and confidence four months after training, and its incorporation of a mixed-method approach, providing a more nuanced understanding of findings and changes therein. However, limitations include the small sample size and the restriction of the training to GPs and practice nurses in a single PHN area. This focus may limit the generalisability of the findings to other PHNs due to differences such as variations in HealthPathways™ utilisation and the availability of dementia rehabilitation programs. However, it is evident in our small sample that a one-off course could produce some initial improvements in knowledge gain, confidence, and attitude to dementia care but was not enough to change real practice in the short term.

Future research should focus on addressing barriers to accessing dementia rehabilitation across multiple levels. Enhancing support for GPs in referring people with dementia to allied health services is crucial, and funding specific dementia rehabilitation programs has shown promise, though further evaluation is needed. There is currently a gap in measuring healthcare professionals’ perceptions of dementia rehabilitation, which is a precursor to changing referral practices. Validating the new dementia rehabilitation scale questionnaire would fill this gap, providing a valuable tool for assessing these perceptions. Additionally, utilising routinely collected GP referral data for allied health services may help monitor the impact of practice changes over time.

## 5. Conclusions

Providing dementia training for GPs and practice nurses with a focus on rehabilitation is an effective strategy to enhance their knowledge, awareness, and confidence in interventions and referrals aimed at reducing dementia-related disability. However, maintaining the benefits of such training may require additional sessions, regular consolidation, or peer mentoring. It is also crucial to address barriers to referrals and implement solutions to overcome these obstacles to improve access to dementia rehabilitation services and support GPs and practice nurses in making these referrals.

## Figures and Tables

**Table 1:** Demographics of dementia training course participants.

	*n* = 17
Age (years)—Mean (SD)	51.9 (9.3)
Gender—*n* (%) - Female - Male	13 (76.5) 4 (23.5)
Professional role—*n* (%) - General practitioner - Practice nurse	14 (82.4) 3 (17.7)
Primary workplace—*n* (%) - General practice clinic - Residential aged care facility - Community health service	13 (76.5) 2 (11.8) 2 (11.8)
Years of practice—Mean (SD)	21.4 (11.5)
Work hours per week—Mean (SD)	29.3 (9.4)
Number of people with dementia treated in the last 12 months—*n* (%) - 1–5 - 6–10 - 11–15 - 16–20 - More than 20 - None	5 (29.4) 5 (29.4) 1 (5.9) 3 (17.7) 3 (17.7) 0 (0)
Undertaken post-graduate training in dementia (Yes)—*n* (%)	1 (5.9)
Know about their Primary Health Network’s Dementia Pathway (Yes)—*n* (%)	4 (23.5)
Referred people with dementia to allied health professionals in the last 12 months (yes)—*n* (%)	10 (58.8)
Allied health professional dementia referrals in the last 12 months—*n* (%) out of the *n* = 10 that responded yes to the prior question - Physiotherapy - Occupational therapy - Speech pathology - Psychology - Exercise physiology - Other (e.g., Podiatry, Dietetics)	9 (90) 8 (80) 5 (50) 5 (50) 4 (40) 6 (60)

**Table 2:** Changes in DKAS, GPACS-D, dementia rehabilitation scale questionnaire, Likert-scaled statements of knowledge and confidence about dementia support, and confidence scale in understanding allied health professional roles for dementia pre- and post-course and follow-up.

	Pre-Course (*N* = 17)	Post-Course (*N* = 17)	Relative Change ^a^ (%)	Absolute Change (Co-Efficient) Robust (95% CI)	Focus Group Participants Post-Course (*N* = 8)	Focus Group Participants at 4 Months (*N* = 8)	Relative Change ^b^ (%)	Absolute Change (Co-Efficient) Robust (95% CI)
DKAS ^c^—Mean (SD)	41.8 (4.2)	46.8 (2.0)	12.1	5.1 (3.2, 6.9) *	47.5 (0.9)	45 (3.5)	−5.3	−2.5 (−5.2, 0.2)
DKAS subscales ^d^—Mean (SD) - Causes and characteristics - Communication and behaviour - Care considerations - Risk and health promotion	10.4 (2.5) 9.1 (1.3) 11.1 (1.6) 11.3 (1.2)	13.6 (1.1) 9.4 (0.9) 11.9 (0.5) 11.9 (0.5)	31.9 3.9 7.4 5.2	3.3 (2.0, 4.6) * 0.4 (−0.4, 1.1) 0.8 (−0.01, 1,7) 0.6 (0.1, 1.1) *	14 (0) 9.5 (0.9) 12 (0) 12 (0)	12.5 (2.3) 9.3 (1.5) 11.8 (0.7) 11.5 (0.9)	−10.7 −2.6 −2.1 −4.2	−1.5 (−3.5, 0.5) −0.3 (−0.9, 0.4) −0.3 (−0.9, 0.4) −0.5 (−1.3, 0.3)
GPACS-D ^e^—Mean (SD)	10.6 (1.4)	11.7 (1.0)	10.1	1.1 (0.4, 1.7) *	12.2 (0.9)	12.6 (0.8)	3.3	0.4 (−0.01, 0.8)
GPACS-D subscales ^f^—Mean (SD) - Attitude to Care - Engagement - Confidence in Clinical Ability	4.3 (0.9) 3.6 (0.9) 2.7 (0.7)	4.7 (0.3) 3.4 (0.7) 3.7 (0.5)	8.1 −7.7 37.2	0.3 (−0.1, 0.8) −0.3 (−0.7, 0.1) 1.0 (0.7, 1.3) *	4.8 (0.3) 3.7 (0.8) 3.8 (0.6)	4.7 (0.4) 4 (0.6) 4 (0.5)	−2.1 9.0 4.5	−0.1 (−0.2, 0.02) 0.3 (−0.01, 0.7) 0.2 (−0.3, 0.6)
Dementia rehabilitation scale questionnaire ^g^—Mean (SD)	68.4 (6.3)	74.8 (4.9)	9.4	6.4 (4.4, 8.5) *	75.3 (4.9)	73.4 (4.6)	−2.5	−1.9 (−5.1, 1.3)
Much can be done to support people with dementia to maintain their independence in everyday activities ^h^—Mean (SD)	4.4 (1.0)	4.8 (0.4)	7.9	0.9 (0.03, 1.7) *	4.8 (0.5)	4.5 (0.5)	−5.3	−1.1 (−3.4, 1.2)
I know which allied health professionals in my area provide therapy for people with dementia to help them maintain their independence for as long as possible ^i^—Mean (SD)	2.1 (0.9)	3.8 (1.0)	77.4	3.1 (1.6, 4.6) *	3.8 (0.9)	3.4 (1.1)	−9.9	−0.8 (−2.2, 0.7)
I feel confident to discuss dementia reablement and rehabilitation therapies with my patients with dementia ^j^—Mean (SD)	2.2 (1.0)	4 (0.4)	78.6	4.9 (1.7, 8.1) *	4 (0.5)	4.4 (0.5)	9.5	1.7 (−0.3, 3.6)
I feel confident my referrals to health professionals will be accepted for people living with dementia ^k^—Mean (SD)	3.1 (1.3)	4.1 (0.7)	32.1	1.6 (0.5, 2.8) *	4.1 (0.6)	4 (1.1)	−3.2	0 (−1.8, 1.8)
Confidence in understanding allied health professional roles for dementia ^l^—Mean (SD)	6.5 (1.5)	8.1 (1.0)	24.9	2.4 (0.8, 3.9) *	7.9 (1.1)	7.9 (0.8)	0	−0.1 (−2.1, 1.9)

^a^ Relative change (%) pre- and post-course =$\;\left(\mathrm{mean}\;\mathrm{post}\;\mathrm{course}\;\mathrm{score}-\mathrm{mean}\;\mathrm{pre}\;\mathrm{course}\;\mathrm{score}\right)÷\mathrm{mean}\;\mathrm{pre}\;\mathrm{course}\;\mathrm{score}\times 100\%$. ^b^ Relative change (%) post-course and follow-up learning activity =$\;\left(\mathrm{mean}\;\mathrm{follow}\;\mathrm{up}\;\mathrm{score}-\mathrm{mean}\;\mathrm{post}\;\mathrm{course}\;\mathrm{score}\right)÷\mathrm{mean}\;\mathrm{post}\;\mathrm{course}\;\mathrm{score}\;\times 100\%$. ^c^ Dementia Knowledge Assessment Scale (DKAS). Each item is scored, with 0 denoting an incorrect response to a factually true or false statement and 2 indicating a correct response. Total score ranges from 0 to 50 (sum of all items). A higher score signifies a more comprehensive understanding of the dementia. ^d^ DKAS comprises four subscales: (a) Causes and characteristics (items 1 to 7), (b) Communication and behaviour (items 8 to 13), (c) Care considerations (items 14 to 19), and (d) Risks and health promotion (items 20 to 25). Subscale scores range from 0 to 12 except for Causes and characteristics 0–14. A higher subscale score (sum of the respective items) signifies a more comprehensive understanding of the subject. ^e^ General Practitioners’ Attitudes and Confidence towards Dementia Survey (GPACS-D). Total score was computed by summing all subscale average scores. Score ranges from 3 to 15. Higher scores signify more positive attitudes and greater confidence regarding dementia care. ^f^ GPACS-D encompasses three subscales: (a) Attitude to Care (AC—items 1, 3, 4, 5, 6, and 9), (b) Engagement (E—items 2, 7, and 8), and (c) Confidence in Clinical Ability (CCA—items 10, 11, 12, 13, 14, and 15). Subscale scores were computed by summing scores (with consideration for reverse-scored items 2, 7, 8, and 10) and dividing by the number of items in the subscale, resulting in an average score out of 5 for each subscale. Subscale average score ranges from 1 to 5. Higher subscale scores signify more positive attitudes and greater confidence regarding the specific aspect of dementia care respectively. ^g^ Dementia rehabilitation scale questionnaire was computed by summing scores for each item while considering reverse-scored items 1, 2, 4, 5, 9, and 13. Scores ranges from 16 to 80. Higher scores signify more positive perception towards dementia rehabilitation. ^h,i,j,k^ Likert scale agreement ratings of 1 to 5 (1 = Strongly agree, 3 = Neither agree nor disagree and 5 = Strongly agree). A higher rating signifies more agreement with the statement. ^l^ Confidence rating in allied health roles ranges from 0 to 10 (0 = I know nothing, 10 = I know very well). A higher score signifies more confidence in understanding the roles of allied health professionals. * *p* value < 0.05.

## Data Availability

Data are available on request from the corresponding author.

## Supplementary Materials

The following supporting information can be downloaded at: https://www.mdpi.com/article/10.3390/nursrep14040226/s1, Table S1: Optional follow-up learning activity.

## Public Involvement Statement

In developing the dementia training program for General Practitioners (GPs) and practice nurses, the research team engaged with individuals living with dementia and their care partners. They held workshops to gather insights and experiences, which helped shape the training content and ensure it meets real-world needs.

## Guidelines and Standards Statement

This manuscript was drafted against the Equator Network reporting guidelines, Lee, S.D.; Iott, B.; Banaszak-Holl, J., et al. Application of Mixed Methods in Health Services Management Research: A Systematic Review, Med Care Res Rev. 2022, 79, 331–344 for mixed methods research.

## Appendix A. Dementia Rehabilitation Scale Questionnaire

Please read the following statements carefully. Once you have read each statement, please circle the number on the scale that corresponds with your answer, where 1 is ‘Strongly Disagree’, 3 is ‘Neither Agree or Disagree’ and 5 is ‘Strongly Agree’.

	**Strongly Disagree**	**Disagree**	**Neither Agree or Disagree**	**Agree**	**Strongly Agree**
1. Rehabilitation is aimed at improving function for people only after a specific acute event like a stroke or a hip fracture	1	2	3	4	5
2. People with dementia are unable to engage in rehabilitation	1	2	3	4	5
3. Allied health professionals can help reduce carer partner stress	1	2	3	4	5
4. Nothing can be done to keep people with dementia engaged in meaningful activities	1	2	3	4	5
5. If a person with dementia is having difficulty with an activity, support should be organised to do it all for them	1	2	3	4	5
6. Interventions should support the person with dementia to continue activities that are meaningful to them	1	2	3	4	5
7. Exercise may slow cognitive decline in people with dementia	1	2	3	4	5
8. Exercise can improve physical function and mobility in people with dementia	1	2	3	4	5
9. The only role of occupational therapy for people with dementia is home safety and driving assessments	1	2	3	4	5
10. It’s important for people with dementia to remain mentally and socially active	1	2	3	4	5
11. Memory and cognitive strategies can support functional activities in the mild to moderate stages of dementia	1	2	3	4	5
12. A combination of exercise and home hazard modifications can reduce risk of falls in people with dementia	1	2	3	4	5
13. The only role of a speech pathologist in dementia is to manage swallowing difficulties	1	2	3	4	5
14. Psychologists can help people with dementia manage grief associated with a diagnosis	1	2	3	4	5
15. Cognitive rehabilitation can improve activities of daily living and maintain relationships for people with dementia	1	2	3	4	5
16. Communication practice, aides and strategies can help support communication for people with dementia and their care partners	1	2	3	4	5
Scoring: Add scores (taking note of the reverse scored items 1, 2, 4, 5, 9 and 13) for each item to derive the total score. A higher total score indicates a better perception towards dementia rehabilitation.					
