# Supplementary material for: Dementia Rehabilitation Training for General Practitioners and Practice Nurses: Does It Make a Difference?
Source: Nurs Rep. 2024 Oct 21;14(4):3108–25. doi: 10.3390/nursrep14040226 (PMC11503384; doi:10.3390/nursrep14040226)
Supplement: Supplementary file 1 [file nursrep-14-00226-s001.zip › nursrep-3185628-supplementary.pdf]

Supplementary material Table S1.

<u>Optional follow-up learning activity</u>

<table>
<tr><td>Topic</td><td colspan="3">Dementia</td></tr>
<tr><td>CPD</td><td>Self-reporting

Case review/analysis</td><td>Estimated duration</td><td>EA – 1 hour (Part 1)

RP– 2 hours</td></tr>
<tr><td>Date</td><td colspan="3">Oct 23-Feb 2024</td></tr>
<tr><td>Aim</td><td colspan="3">To assist general practitioners, reflect on their approach to dementia care and referrals</td></tr>
<tr><td>Learning outcomes</td><td colspan="3">• Be able to assess the 5 domains of the dementia framework and devise an appropriate care plan.

• Build familiarity with dementia resources to assist people with dementia and their carers/family

• Build knowledge of allied health professionals with an interest in dementia in your local area</td></tr>
</table>

Instructions
**Part 1**<br><br>• Familiarise yourself with the SEMPHN dementia pathway website: Dementia Pathways (semphn.org.au) – Read the first five steps for the person living with dementia and for their carer/family member by clicking on the relevant section.<br><br>• Review the Forward with Dementia General Practice referral flow chart for medical and psychosocial support after a diagnosis dementia pathway part 2 concepts (forwardwithdementia.au)<br><br>• Search your area for the nearest physiotherapist, speech pathologist and occupational therapist with an interest in dementia, gerontology or neurology. Private providers can be found through professional association websites:<br>○ Occupational Therapy Australia - Find an Occupational Therapist (otaus.com.au)<br>○ Find a Speech Pathologist (speechpathologyaustralia.org.au)<br>○ https://choose.physio/find-a-physio<br><br>• Can your patients access the dementia rehab program run by Encara [New Dementia Rehabilitation Program for Mornington Peninsula and Casey residents (semphn.org.au)]<br><br>• Do local community health or rehabilitation services see people with dementia?<br><br>• Review the care plan example for Anna [Appendix A]<br><br>**Part 2**

- Find 3 patients who have a diagnosis of dementia in your practice and review what rehabilitation they have been offered (or see a new patient and start with point 2)
- Invite them in to do (or review) a GPMP plan and Team care arrangement based on the five dementia domains.
- Determine patient goals and offer resources and referrals as needed.
- What funding pathways could they use to access allied health? E.g. public, private, GPMP and team care plan, MyAgedCare, NDIS; Encara's dementia program?

Part 3

Having completed the above activities, reflect on:

- What you did differently after completing the learning activities?
- How did you find the process?
- What helped you?
- What stopped you?
- Did the person with dementia and their care partner find this useful?
- Would you feel confident to do this as part of post diagnostic care in the future?
- What other training, resources or services would you need?

____
____
____
____
____
____
____
____
____
____
____
____
____
____
____
